# Exit Meta-Analysis on the Effect of HIV on COVID-19 Mortality, Hospitalization, and ICU Admission

**DOI:** 10.3390/medsci13040261

**Published:** 2025-11-07

**Authors:** Lubna A. Zar, Shahd Hamran, Izzaldin Alremawi, Mohamed Elahtam, Asmaa Abdelmaksoud, Rida Arif, Tawanda Chivese

**Affiliations:** 1 Department of Population Medicine, College of Medicine, QU Health, Qatar University, Doha P.O. Box 2713, Qatar; lz2005357@qu.edu.qa (L.A.Z.); sh2004556@qu.edu.qa (S.H.); ia2206734@qu.edu.qa (I.A.); me1901983@qu.edu.qa (M.E.); 2 Hamad Medical Corporation, Doha P.O. Box 3050, Qatar; aa1602684@qu.edu.qa (A.A.); ra1605167@qu.edu.qa (R.A.); 3 Department of Science and Mathematics, School of Interdisciplinary Arts and Sciences, University of Washington Tacoma, Tacoma, WA 98402, USA

**Keywords:** exit meta-analysis, HIV, COVID19, mortality, hospitalization, intensive care services

## Abstract

**Purpose:** The COVID-19 pandemic has led to the publication of numerous primary studies and meta-analyses; however, conclusive evidence on whether HIV infection influences COVID-19 outcomes among people living with HIV (PLHIV) is still lacking. This research uses a novel technique, the *exit meta-analysis*, to conclusively update the evidence of HIV’s impact on COVID-19-related mortality, hospitalization, and need for Intensive Care Unit (ICU) admission in severe disease. **Methods:** A search of PubMed, EMBASE, Cochrane Reviews (CDSR), SCOPUS, CINAHL reviews and Google Scholar databases was conducted up to the 18 January 2024 for meta-analyses and observational studies that reported adjusted associations for the effect of HIV on COVID-19 related mortality, hospitalization, and ICU admission. Evidence from existing meta-analyses was summarized narratively, and an updated meta-analysis was carried out using a bias-adjusted inverse variance heterogeneity model. Subgroup analysis was carried out for age groups and geographical regions. **Results:** Of 3153 records identified, 20 meta-analyses and 56 primary studies, with a total of 27,936,428 participants, including 655,882 PLHIV, were included. A review of the meta-analyses showed conflicting results for all outcomes. In the updated synthesis, HIV was associated with higher odds of mortality (aOR 1.43, 95% CI: 1.01–1.86, I^2^ = 90.7%) and ICU admission (aOR 1.49, 95% CI: 0.67–2.30, I^2^ = 88.8%), but not hospitalization (aOR 1.11, 95% CI: 0.78–1.48, I^2^ = 97.5%). The results for both ICU admission and hospitalization include the null value, leading to lower certainty. The exit meta-analysis suggested conclusive results for mortality (DAts score = −0.012) and hospitalization (DAts score = −0.014), but not for ICU admission. **Conclusions:** This exit meta-analysis provides conclusive evidence that HIV increases mortality in people with COVID-19; however, more studies may be required to address ICU admission and hospitalization.

## 1. Introduction

The exponential growth in scientific publications has raised substantial concerns about research redundancy and waste, particularly in biomedical science, where an estimated 85% of research investment may be wasted due to avoidable flaws in study design, conduct, analysis, and reporting [1,2]. A major contributor to this is the proliferation of studies that fail to address knowledge gaps, often duplicating prior work without introducing novel methods or addressing different contexts [3,4]. Applying this lens to the rapidly expanding literature on HIV status and COVID-19 outcomes—such as mortality, hospitalization, and intensive care unit (ICU) admission—offers a timely opportunity to evaluate whether the existing evidence base has reached saturation or if further research is justified. Systematic prioritization frameworks, including those in The Lancet series on increasing value and reducing waste, emphasize the importance of grounding new research on prior systematic reviews and clearly defined knowledge gaps to avoid redundancy and inform clinical and public health decisions [4,5].

As of 2024, more than 20 meta-analyses have been published to answer the question regarding the effect of HIV on adverse COVID-19 outcomes, with conflicting findings. Results from observational studies have also been conflicting, andmany studies continue to be published after the last meta-analysis [6,7,8,9,10]. This has raised valid concerns about research waste [11]. A key concern is that current meta-analysis research methods have lacked techniques to evaluate the conclusiveness of research on a particular topic. Such techniques may minimize research waste as researchers will be able to assess whether the available evidence is conclusive or if it requires updating by conducting new primary studies or new evidence syntheses [4]. A recently developed method, the *exit meta-analysis*, has been proposed to provide a way to determine the conclusiveness of a meta-analysis [11]. This method evaluates whether accumulating evidence on a specific question has reached a stable conclusion or whether new primary or secondary studies are still warranted. However, this technique has not yet been applied in research beyond its original validation studies.

COVID-19 remains a global threat since its emergence in late 2019 [12]. Identifying susceptible individuals to adverse clinical outcomes of COVID-19, such as hospitalization and death, should be prioritized to provide earlier prevention [13,14]. People living with HIV (PLHIV) may be particularly vulnerable, though the evidence on their risk remains inconsistent and debatable [13,14]. HIV may worsen clinical outcomes of COVID-19 because chronic immunosuppression in PLHIV contributes to an impaired immune response against SARS-CoV-2 and may hinder vaccine effectiveness [15]. On the other hand, anti-retroviral therapy (ART) may have benefits in reducing the morbidity from COVID-19 [16]. Notably, the disproportional distribution of HIV in low- and middle-income countries, which are home to more than two-thirds of PLHIV, combined with low COVID-19 vaccination rates, may increase the vulnerability of PLHIV to adverse COVID-19 clinical outcomes [17]. Given the uncertainty, there is a need to synthesize all the evidence concerning the effect of HIV on COVID-19 outcomes.

Despite the publication of several primary and secondary research studies, it is still not clear whether HIV increases vulnerability to worse outcomes in individuals with COVID-19. Systematic reviews and meta-analyses [13,14,18,19,20,21,22,23,24,25] have produced conflicting results. Some meta-analyses have found no effect of HIV on COVID-19-related mortality [13,21,22,24,25]. Other meta-analyses have reported significant associations between HIV and worse morbidity and mortality outcomes of COVID-19 [13,14,18,19,20,21,22,23,24,25]. A possible explanation for the heterogeneity in the results of the meta-analyses is the diversity in the study populations and population-level vulnerability to adverse COVID-19 outcomes. For example, European populations, which are generally older, experienced higher COVID-19 mortality rates compared to the younger populations in Asia, Africa, and South America [26]. Another reason could be the differences in methodology and the reliance on unadjusted effect sizes, which may have led to confounded findings.

To provide a conclusive assessment of the association between HIV and adverse outcomes of COVID-19, we applied the novel *exit meta-analysis* technique. This approach was used to evaluate the conclusiveness of evidence on the effect of HIV on COVID-19-related mortality, hospitalization and ICU admission. We also updated the current evidence on the impact of HIV on COVID-19 morbidity and mortality by reviewing existing meta-analyses and conducting a new meta-analysis of observational studies with adjusted effect estimates.

## 2. Methods

### 2.1. Study Design and Protocol Registration

This study is a meta-review of existing meta-analyses and an updated meta-analysis of observational studies. This study was conducted according to the Preferred Reporting Items for Systematic Reviews and Meta-Analysis (PRISMA) guidelines, ref [27] and the protocol was registered with the international database of prospectively registered protocols of systematic reviews and meta-analyses (CRD42020221311) [28], Registration PROSPERO: CRD42020221311 https://www.crd.york.ac.uk/prospero/display_record.php?RecordID=221311 accessed on 24 September 2025).

### 2.2. Search Strategy and Data Sources

We searched PubMed, EMBASE, Cochrane Database of Systematic Reviews (CDSR), SCOPUS, CINHAL reviews and Google Scholar up to 18 January 2024. We manually screened and conducted citation searches of the top 20 related articles of the included studies. The search strategy combined Medical Subject Headings (MeSH) terms and keyword terms related to COVID-19 and HIV infection. For example, in PubMed, terms used for COVID-19 included: (“COVID-19” OR “SARS-CoV-2” OR “novel coronavirus” OR “coronavirus disease 2019” OR “Wuhan pneumonia”), and for HIV: (“HIV” OR “human immunodeficiency virus” OR “AIDS” OR “acquired immunodeficiency syndrome”). The full electronic search strategy is given in Supplementary Document S1.

### 2.3. Screening of Studies for Inclusion

Study records retrieved from the searches were exported into EndNote 21 referencing software for deduplication and then exported into the Rayyan platform [29] for initial screening by title and abstract independently by two pairs of reviewers (L.Z., S.H., M.E. and I.A.). The studies included after initial screening were then assessed for eligibility using full text, and any conflicts were resolved through discussion, and if unresolved, by consulting TC.

### 2.4. Eligibility

For the meta review, meta-analyses reporting on the effect of HIV on COVID-19 clinical outcomes in people with confirmed COVID-19 were included. Literature and systematic reviews without meta-analyses were excluded. For the updated meta-analysis, we included observational studies with adjusted effects of HIV on COVID-19 clinical outcomes. Studies which did not report on the outcomes of this review, case reports, case series, duplicate publications, studies with participants who were clinically diagnosed with COVID-19 (without molecular confirmation), and reviews were excluded.

### 2.5. Outcomes

The primary outcome was mortality in individuals with COVID-19, measured as all-cause mortality, 28-day mortality, 30-day mortality or COVID-19-specific mortality. Secondary outcomes of interest were hospitalization and the need for ICU services for COVID-19, defined as transfer to the ICU, intubation and/or the need for mechanical ventilation.

### 2.6. Data Extraction

Two authors independently extracted data from the included studies. From each included meta-analysis, we extracted data on authors, year of study, number of included studies, outcomes assessed, model/s used, and overall effect sizes for each outcome. For the updated meta-analysis, we extracted study characteristics such as author, year of publication, study design, country, sample size, and the number of participants with COVID-19, if the number is different from the sample size. We extracted adjusted effect sizes and their 95% confidence intervals (95% CIs) for the effect of HIV on the following outcomes: hospitalization for COVID-19, ICU and mortality.

### 2.7. Assessment of Study Quality

Two reviewers independently assessed the quality of the primary studies across safeguards within seven domains using the MethodologicAl Standard for Epidemiological Research (MASTER) scale [30]. The MASTER scale consists of seven standards, which are: equal recruitment, equal retention, equal ascertainment, equal implementation, equal prognosis, sufficient analysis and temporal precedence. The total number of safeguards within these domains is 36, and higher scores indicate higher study quality. Any disagreements between the reviewers was resolved by discussion.

### 2.8. Synthesis of Findings

To avoid analyzing overlapping studies from previous meta-analyses, a descriptive narrative analytical approach was used for the meta-review. The narrative synthesis of the included meta-analyses was carried out using tables and descriptions of effect sizes for each outcome and summarizing the overall evidence. For the updated meta-analysis, the characteristics of the included studies were described narratively. We used a bias-adjusted inverse heterogeneity model (the quality effects model) to carry out meta-analysis of adjusted effect sizes for each outcome [31]. This quality effects model assumes that there is one true effect size shared by all included studies, and any deviations from this are due to random and/or systematic error [31]. The quality effects model allows for the weighting of study quality in meta-analysis models. In the quality effects model, the overall study weights were computed based on inverse variance, heterogeneity and study quality. The MASTER scale scores were normalized to a 0–1 range and used to adjust individual study weights, giving greater influence to studies with stronger methodological safeguards. In *Metan,* this process is automated by specifying the quality effects (QE) model and quality variable model in the analysis code. After meta-analysis, we displayed the effect sizes of each outcome of hospitalization, intensive care services and mortality in PLHIV compared to individuals without HIV, from each study using forest plots. Results were presented in forest plots, with overall effect sizes and their 95% confidence intervals (95%Cis) reported, stratified by the effect size type (adjusted odds ratio: aOR, adjusted hazard ratio: aHR, adjusted relative risk: aRR). Heterogeneity was assessed using the I^2^ statistic, defined as low, moderate or high, based on cut-offs of 25%, 50%, 75%, respectively, with Cochran’s Q *p*-value serving as a confirmatory test of significance [32]. Doi plots and the LFK index, funnel plots and Egger’s test were used to assess publication bias. An LFK index > 1 or <−1 suggests asymmetry and a higher chance of publication bias. We conducted a leave-one-out sensitivity analysis to assess the robustness of our findings by sequentially excluding each study and observing its impact on the pooled results.

### 2.9. Subgroup Analysis

We pre-specified subgroup analysis for the region of origin of the studies (Africa, Europe, North America and South America) and for age groups, using the broad categories of age < 60 years and ≥60 years. Data for heterogeneity by age group for the COVID-19 ICU outcome were not reported by any included study. Regions were selected based on their relevance to HIV and COVID-19 care.

### 2.10. Exit Meta-Analysis

The exit meta-analysis is a novel methodological technique developed with the intention of determining whether the current available evidence for a specific research question is sufficient and requires no further studies. The conclusiveness of current evidence was assessed using the Doi-Abdulmajeed Trial Stability (DAts) index, which is a convergence index computed in the exit meta-analysis module in Stata (*exitma*) [11]. The exitma module is used to conduct cumulative meta-analyses (CMA), and the final meta-analytic effect size is compared to the CMA effect size, which generates the ∆Ci index. The DAts index is the variance of the ∆Ci index examines the exit status or conclusiveness of a meta-analysis by assessing the stability of an effect measure as sequential studies are added to the analysis. The convergence plots generated illustrate the changes in ∆Ci index as each study is added to the CMA in a temporal sequence, and the vertical dotted line indicates the point at which 50% of the total participants from all the studies are included in the CMA. A meta-analysis is classified as an exit meta-analysis, indicating no need for updates or future studies on the question. If the DAts values are less than 0, DAts values between 0 and 0.05 suggest a potential exit status, and DAts values greater than 0.05 imply that the analysis currently is not conclusive, i.e., not an exit meta-analysis. If a meta-analysis is determined as an “exit” meta-analysis, this implies that it is highly unlikely that the conclusions of the meta-analysis will be changed by the addition of new studies. Stata version 18.0 [33] was used for all analyses.

### 2.11. Ethics

This research used data from published studies; therefore, no ethical approval was required.

## 3. Results

### 3.1. Search Results

**Figure 1:**
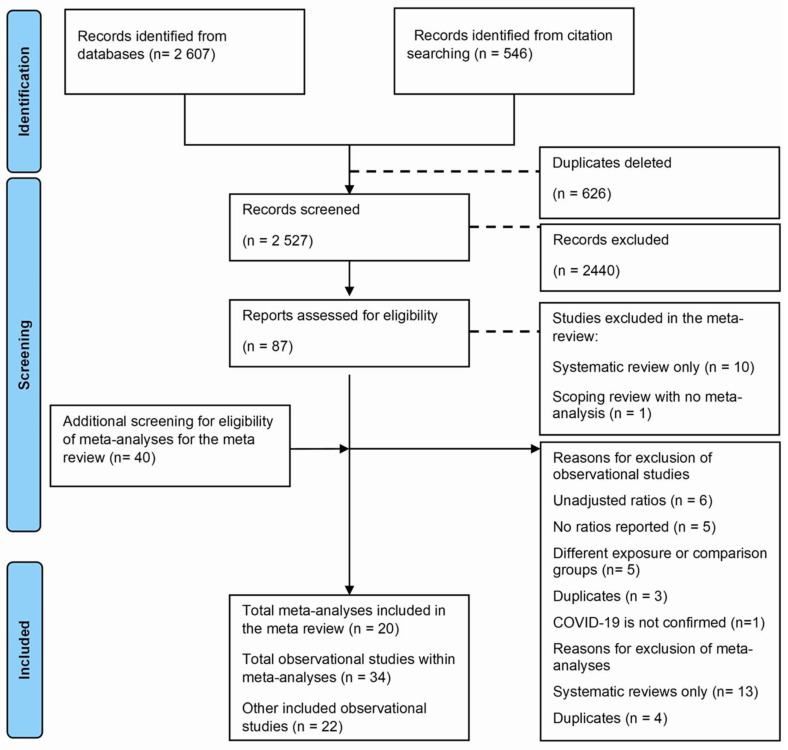
Flowchart for the meta-review and updated meta-analysis.

A total of 3153 studies were identified from databases and citation searching, of which 625 were duplicated and deleted. During screening using titles and abstracts, 2440 studies were excluded. Following full-text screening of 87 studies, 29 were excluded, and 20 meta-analyses and 38 observational studies were included, of which 17 were within these meta-analyses. We screened these meta-analyses for other observational studies and included 18 more, with 56 observational studies. The reasons for exclusions using full text were as follows: reviews without meta-analyses (n = 11), primary studies without adjusted effects (n = 6), with additional reasons described in Figure 1.

### 3.2. Characteristics and Results of Previous Meta-Analyses

Initially, 22 systematic reviews and meta-analyses were identified; of those, a total of 20 meta-analyses [13,14,18,19,20,21,22,23,24,25,34,35,36,37,38,39,40,41,42,43] were included (Supplementary Table S1). Two studies were excluded as they were only systematic reviews without meta-analyses [44,45]. The meta-analyses were published between the years 2020–2023. All 20 studies used the random effects model for the meta-analysis, without accounting for study quality in the weighting process. In total, all 20 meta-analyses reported on mortality, only two meta-analyses reported on hospitalization [13,35], and two reported on ICU admission [14,20,43]. The number of included studies ranged from two studies in one meta-analysis [13,20] to 84 studies in another [38]. Out of the 20 meta-analyses, 16 had unadjusted analyses, and the remainder carried out analyses of adjusted effect sizes.

Most of the studies (n = 15) had findings that suggested a moderate increase in the risk or odds of mortality in PLHIV compared to people without HIV with overall effect sizes ranging from an OR of 0.81 (95% CI 0.47–1.41) in one meta-analysis of unadjusted effects with 23 studies [13] to an OR of 1.95 (95% CI 1.62–2.34) in another meta-analysis of adjusted effects with 5 studies [20] (Supplementary Table S1). For hospitalization, two previous meta-analyses showed a rise in PLHIV compared to no HIV, with overall effects sizes ranging from an OR of 1.49 (95% CI 1.01, 2.21) in one meta-analysis of unadjusted effects with 6 studies to an OR of 1.67 (95% CI 0.76, 3.71) in another meta-analysis of unadjusted effects with three studies [35] (Supplementary Table S1). Overall, the effect of HIV on COVID-19 ICU admission ranged from an RR of 1.24 (95% CI 1.08, 1.41) in one meta-analysis of unadjusted effects with 4 included studies [14] to an RR of 1.50 (95% CI 0.84–2.67) in another meta-analysis of adjusted effects with two studies.

### 3.3. Characteristics of Included Primary Studies

A total of 56 primary studies were included in the updated meta-analysis, and most were retrospective cohorts, with the exception of four case–control studies [46,47], two cross-sectional studies [48,49,50,51], and two prospective cohort studies [10,52] (Supplementary Table S2). Most studies were conducted in North America (n = 25), followed by Africa (n = 14), then Europe (n = 12). The main covariates which studies adjusted for were age, sex, and comorbidities. Participant ages ranged from a mean of 37 years in one study from Mexico [53] to a median age of 69 (54–79) in another study from England [54].

### 3.4. Quality Assessment of Primary Studies

The MASTER scale score ranged between 20 and 28 out of 36, with an average score of 24, suggesting that these studies were of moderate quality. Most studies scored lower in the ‘equal ascertainment’ domain due to the absence of blinding, and the ‘temporal precedence’ domain, due to the retrospective design of the studies and the unavailability of data regarding the duration of HIV status (Supplementary Figure S1).

### 3.5. Effect of HIV on COVID-19 Mortality

**Figure 2:**
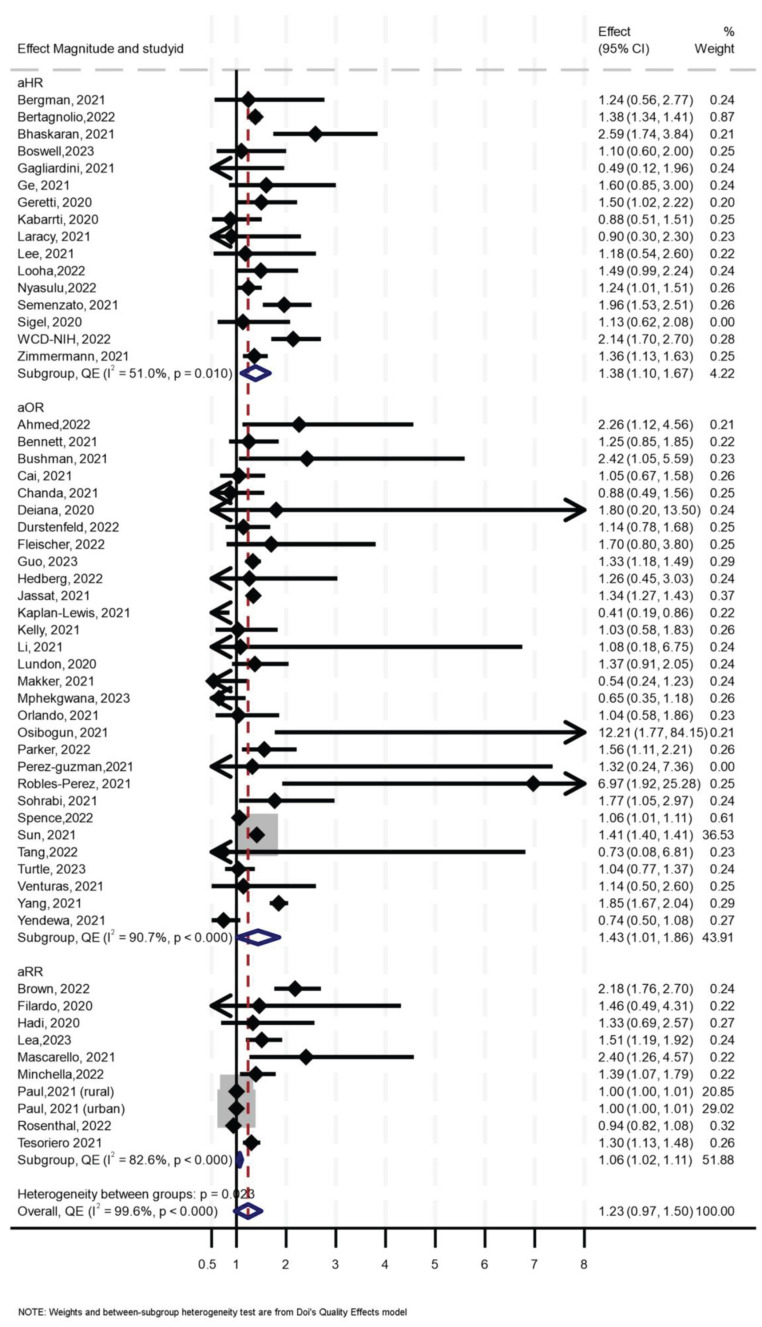
Forest Plot for the effect of HIV on COVID-19-related mortality.

**Figure 3:**
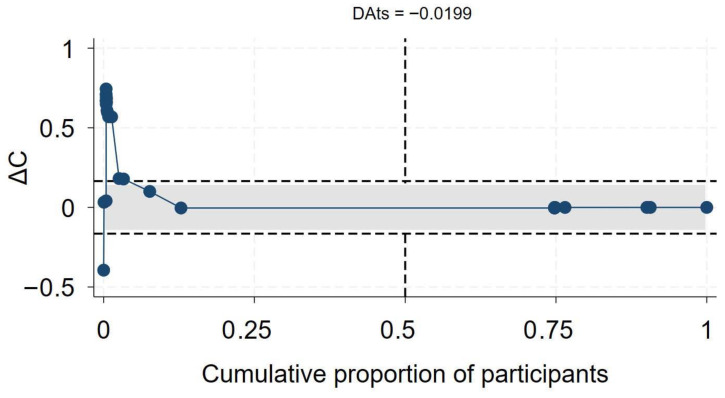
Exit meta-analysis convergence plot and DAts index for the association between HIV and COVID-19 related mortality.

Of the 56 primary studies, 55 studies, with a total sample size of 27,936,428, of which at least 655,882 were PLHIV, reported data on mortality. The effect sizes ranged from an aOR of 0.41 (95% CI 0.19–0.86) [55] to an aOR of 12.21 (95% CI 1.77–84.15) [56] (Figure 2). The synthesis of hazard ratios (n = 17 studies) showed a 39% increase in the hazard of mortality in PLHIV (aHR 1.39, 95% CI 1.10–1.67) with moderate heterogeneity (I^2^ = 51.4%) (Figure 1). In the analysis of studies with adjusted OR (n = 30 studies), the odds of mortality were 45% higher in PLHIV compared to those without HIV (aOR 1.44, 95% CI 1.01–1.86), with high heterogeneity (I^2^ = 90.7%) (Figure 1). The synthesis of studies that used aRR showed a small increase in the risk of mortality in PLHIV compared to those without HIV (aRR 1.07, 95% CI 1.02–1.11, I^2^ = 82.6%, n 9 studies) (Figure 1). Both the convergence plot (Figure 3) and the DAts index of 0.02 indicated that the results were conclusive for the association between HIV status and COVID-19-related mortality.

Sensitivity analysis using leave-one-out (Supplementary Figure S2) showed two influential effect sizes, both reported in one study [57], and analysis without them showed consistent results similar to the aforementioned. Funnel and Doi plots, along with an LFK index of 2.94, indicated asymmetry, suggesting possible publication bias (Supplementary Figure S3). In subgroup analysis, the effect of HIV on mortality was slightly higher (aOR 1.37, 95% 0.08–1.93, I^2^ = 36%) in those aged > 60 years compared to the effect of HIV in those aged ≤ 60 years (OR 1.15, 95% CI 0.13–2.17, I^2^ = 54%), although the analysis suggested a need for more data (*p* for interaction = 0.26) (Table 1).

In further stratified analysis, for studies in North America, synthesis of studies which reported aOR suggested that HIV was associated with higher odds of mortality by 39% (aOR 1.39, 95% CI 0.91–1.86, I^2^ = 95.4%) while the synthesis of studies which reported aHR suggested no effect (aHR 1.04, 95% CI 0.68–1.40, I^2^ = 0.0%, *p* < 0.668). For Sub-Saharan Africa, synthesis of studies which reported aOR suggested that HIV was associated with 40% higher odds of mortality (aOR 1.40, 95% CI 0.81–1.99, I^2^ = 54.6%) while synthesis of studies which reported aHR suggested that HIV was associated with 35% higher hazards of mortality (aHR 1.35, 95% CI 0.65–2.06, I^2^ = 78.8%). For Europe, a synthesis of the studies reporting the aOR suggested no effect of HIV on COVID-19 mortality (aOR 1.135, 95% CI 0.81–1.44, I^2^ = 0%), but studies reporting aHR showed a 75% increase in hazards of mortality (aHR 1.75, 95% CI 1.32–2.18, I^2^ = 31.3%). There were only two studies from Asia, and both showed that HIV was associated with higher odds and hazard of mortality (Table 2). The three studies from South America also suggested that HIV was associated with a higher risk and odds of COVID-19-related mortality (Table 2).

### 3.6. Effect of HIV on COVID-19-Related ICU Admission

**Figure 4:**
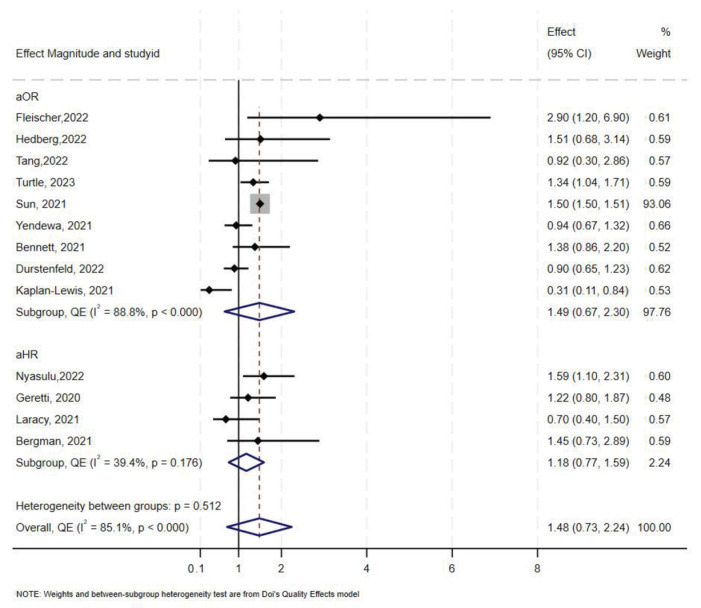
Forest plot for the effect of HIV on COVID-19-related ICU admission.

Overall, 16 studies [10,47,52,55,58,59,60,61,62,63,64,65,66,67,68,69], reported data on COVID-19-related ICU admissions among PLHIV compared to those without HIV. Five of the studies reported aHR, nine reported aOR, and only two studies reported aRR. In overall synthesis, HIV status was associated with higher odds (aOR 1.49, 95% CI 0.67–2.30) with high heterogeneity (I^2^ = 89.0% *p* < 0.001), and higher hazards of ICU admission (aHR 1.39, 95% CI 0.69–2.10) with high heterogeneity (I^2^ = 77.7%, *p* < 0.001) (Figure 4). Of the two studies that reported aRR, one reported an aRR of 0.85 (95% CI 0.74–0.98) and the other an aRR of 3.34 (95% CI 1.98–5.62) [47,62]. Exit status could not be determined as one study contributed over 50% of the total sample size.

The Doi plot and funnel plot (Supplementary Figure S4) indicated minor asymmetry. Sun et al. (2021) [63] contributed to 92.8% (sample size: 1,446,913) of the total weight. Leave-one-out analysis showed that the aOR approached the null value when Sun et al. was removed (aOR 1.07, 95% CI 0.73–1.41) (Supplementary Figure S5).

In stratified analysis, the estimated effect of HIV on ICU admission was a 46% increase in odds (aOR 1.46, 95% CI 0.47–2.45, I^2^ = 94%, *p* < 0.000) in North America, and a 1.36 increase in odds in Europe (Table 2). Only two studies reported COVID-19-related ICU admission in sub-Saharan Africa. Fleischer et al. [66] reported an aOR of 2.90 (95% CI 1.20–6.90) and Nyasulu et al. [66] reported a lower aOR of 1.59 (95% CI 1.10–2.31). One study from South America reported an aRR of 3.34 (95% CI 1.98–5.62).

### 3.7. Effect of HIV on COVID-19-Related Hospitalization

**Figure 5:**
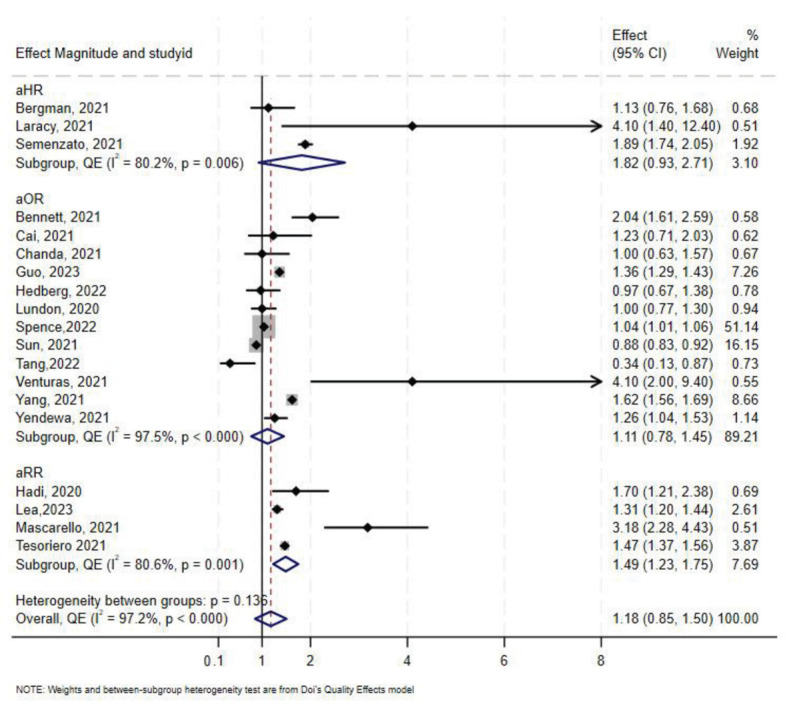
Forest plot for the effect of HIV on COVID-19-related hospitalization.

**Figure 6:**
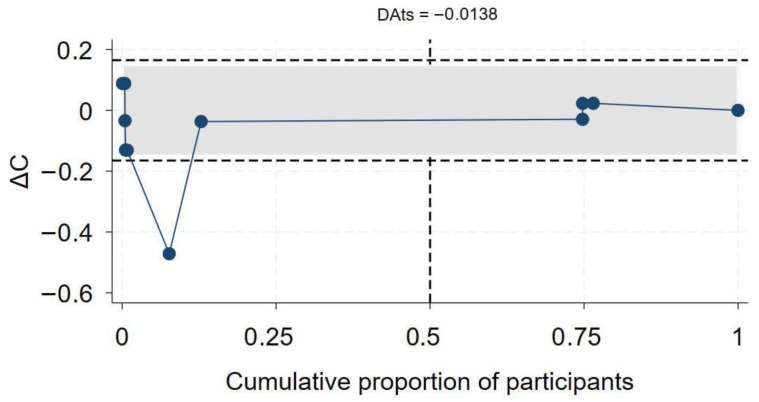
Exit meta-analysis convergence plot and DATs index for the association between HIV and COVID-19 hospitalization.

A total of 19 studies [7,46,47,50,60,61,63,64,65,68,69,70,71,72,73,74,75,76,77,78] assessed the association between HIV status and COVID-19-related hospitalization. Twelve studies reported aOR, three reported aHR, and the remaining four reported aRR. In the overall synthesis of studies reporting the aHR, HIV was associated with a higher hazard of hospitalization by almost two-fold (aHR 1.82, 95% CI 0.93–2.71, I^2^= 80.2%). A synthesis of studies reporting the aRR showed that HIV was associated with a 49% increase in risk of hospitalization. However, in studies reporting the aOR, the overall synthesis did not show an effect of HIV on the odds of hospitalization (Figure 5). The convergence plot and the DAts index suggested conclusive evidence for the studies reporting the aOR for the association between HIV-status and hospitalization (DAts index = −0.0138) (Figure 6). However, it was not possible to conduct an exit meta-analysis for aHR and aRR due to the limited number of available studies.

The Doi and funnel plot showed symmetry, suggesting no evidence of publication bias (Supplementary Figure S6). Sensitivity analysis revealed that Spence et al. [72] contributed 56.7% of the total weight in the meta-analysis; excluding this study resulted in an overall odds-ratio of 1.28 (aOR 1.28, 95% CI 0.93–1.62) (Supplementary Figure S7).

In stratified analysis the effect of HIV on hospitalization was minimal (aOR 1.09, 95% CI 0.75–1.43, I^2^ = 98%, *p* < 0.0001) in North America, and a 31% increase in odds of hospitalization in Europe (aOR 1.31, 95% CI 0.21–2.42, I^2^ = 92%, *p* < 0.00) in Europe (Table 2). Only one study reported results for hospitalization in sub-Saharan Africa [50], which showed no association between HIV and COVID-19 with an aOR 1.00 (95% CI 0.63–1.57). As for South America, only one study was available, and they reported an aRR of 3.18 (95% CI 2.28–4.43) [47].

## 4. Discussion

This meta-review and updated meta-analysis revealed conflicting findings in existing meta-analyses regarding the effect of HIV on clinical outcomes of COVID-19. For mortality, previous meta-analyses have found effects ranging from protective to detrimental. Findings from existing meta-analyses were fewer and even more uncertain for the outcomes of hospitalization and ICU. In the updated meta-analysis, we found that HIV was associated with increases in hazards, risks and odds of the three outcomes. We observed significant heterogeneity across studies in the effect of HIV on all three outcomes, suggesting that HIV may have varying impacts across populations and health systems. The exit meta-analysis indicated that the current evidence on the association between HIV and mortality may be conclusive. However, the exit meta-analysis suggested a need for more studies on hospitalization and ICU outcomes.

In the current updated meta-analysis of adjusted effects, HIV increased the hazards and odds of mortality by 39% and 44%, respectively. The hazard ratio finding implies that, after adjusting for confounders, HIV was associated with a 39% higher risk of death over time, while the odds ratio suggests a 44% higher likelihood of death. Collectively, these results indicate that PLHIV face a substantially increased risk of COVID-19 mortality, both over time and in aggregate. In the previous meta-analyses, the effect of HIV on mortality rates ranged from an OR of 0.80 to an OR of 2.07. Our results are consistent with findings from the largest existing meta-analyses [18,23], which used adjusted effect sizes, albeit these meta-analyses had fewer studies (a maximum of 40) compared to the current meta-analysis (56 studies). Therefore, the current meta-analysis is the most up-to-date, which includes the totality of available evidence on the effect of HIV on mortality due to COVID-19. The increased mortality risk shows that people living with HIV are more vulnerable to severe COVID-19, even after accounting for other factors. Clinically, these findings underscore the importance of targeted management and prioritization in vaccination and treatment, while from a public health perspective, the findings support integrating HIV and COVID-19 care to reduce excess deaths in this population.

There are several suggested mechanisms by which HIV may contribute to worsened COVID-19 prognosis, including hospitalization, a need for intensive care services and mortality. These mechanisms fall into two broadly categories: biological and social. Lymphopenia as a result of chronic HIV infection leads to an impaired immune response [79]. For instance, CD4 counts <200 were shown to have a three-fold increase in the hazard of severe COVID-19 outcomes among PLHIV [80]. Another major biological factor is the cumulative effect of co-morbidities, including tuberculosis (TB), which is prevalent in low socioeconomic countries in sub-Saharan Africa and Asia. TB further exacerbates the compromised immune system in PLHIV [81]. Social factors, includingisolation during COVID-19 and HIV-related stigma, may reduce adherence to ART and result in increased morbidity and mortality [82]. Recent findings show that social stigma and economic disadvantages in under-resourced communities lead to health disparities in COVID-19 prevention and treatment, which reduce the quality of life of PLHIV [83].

Our findings indicate an elevated risk of COVID-19-related hospitalization among PLHIV. A prior meta-analysis [13], which included six primary studies, reported an unadjusted OR 1.49 (95% CI 1.01–2.21). A more recent study [35] included three studies that reported on hospitalization and found an OR 1.67 (95% CI 0.76–3.71).

In subgroup analysis for all outcomes, the overall mortality was highest in Asia, although this was based on a few studies. South America, sub-Saharan Africa, and Europe had a similar effect size. Except for North America, the findings in all the other regions generally mirrored those of our main findings, i.e., an elevated risk of worse COVID-19 prognosis for PLHIV when compared to individuals without HIV. Despite the widespread availability of effective COVID-19 vaccines, where mortality due to COVID-19 is low, these findings suggest a higher risk for hospitalizations and consequent higher utilization of health resources for PLHIV during COVID-19 outbreaks. This will have negative implications for high-burden HIV countries in Sub-Saharan Africa, such as South Africa and may likely be worsened by the high prevalence of undiagnosed chronic comorbidities such as metabolic syndromes and cardiovascular diseases [13]. There is a need to prioritize prevention, including prioritizing vaccine access, care and treatment of COVID-19 in PLHIV as recent studies have shown sufficient humoral response to vaccination regardless of low CD4 count [84]. Recent studies have shown that PLHIV are vulnerable to higher rates of severe breakthrough infection with CD4 count <350 cells/mm [85]. Therefore, there is a need for booster vaccinations among PLHIV. A recent meta-analysis found that COVID-19 booster vaccination reduced the risk of COVID-19 infection, hospitalization and death compared to primary vaccination alone in PLHIV [86].

In subgroup analysis by age, we found that the effect of HIV on mortality was worse among individuals >60 years of age, partly due to immunocompromise associated with ageing, and a higher propensity for comorbid diseases, which worsen the COVID-19 severity.

We found only two other systematic reviews which reported data on ICU admission. The first [43] included only one study. The other meta-analysis identified two studies which reported on ICU admission and reported an overall RR of 1.50 (95% CI 0.84–2.67) [20]. In addition to the sparseness of data within these meta-analyses, neither account for confounders when reporting effect measures, mainly due to the limitations of the primary data. Nevertheless, in our meta-analysis, we included covariate-adjusted data from 16 studies, and the results suggested an overall 39% increase in hazard and 49% increase in odds of COVID-19 ICU admission. These hazard and odds reflect patterns similar to those observed for mortality, with ICU admission often serving as a proxy for severe disease. The implications are similar to those discussed regarding mortality.

Our study has several limitations. As the studies included in this synthesis are observational studies, causal inferences should be interpreted with caution. The lack of data from the studies regarding subgroups, such as those taking ART in HIV or CD4+ count, limited the sensitivity analysis in this meta-analysis. Furthermore, subgroup analysis based on gender was rarely reported, even though gender is a known prognostic factor for all-cause mortality among PLHIV [87]. A key strength of this study is its rigorously design and conduct using the updated PRISMA guidelines for systematic reviews and meta-analyses. In addition, we used adjusted effect sizes from studies in the synthesis of evidence, thereby reducing the role of confounding, one of the main weaknesses of previously published meta-analyses. Additionally, this meta-analysis included only studies with laboratory-confirmed COVID-19 or rapid antigen testing, reducing the risk of selection bias. Additionally, we used a quality-adjusted effects model to consider the quality of included studies, which has not been done in any of the previous meta-analyses investigating HIV and COVID-19 outcomes. Finally, we applied the exit meta-analysis technique to examine the conclusiveness of the current literature on the topic at hand.

The exit meta-analysis method has some limitations, including sensitivity to between-study heterogeneity, which can reduce the likelihood of declaring an “exit” and affect the stability of its estimates. The method is model-dependent, having been developed withina common-parameter framework (using IVhet or quality-effects estimators), and its operating characteristics may differ when standard random-effects models are used, limiting straightforward application across many published meta-analyses. Furthermore, it defines conclusiveness only in statistical terms, without considering evolving real-world factors such as new variants or interventions. Nonetheless, it provides a valuable quantitative framework to identify when evidence has stabilized, helping to minimize redundant research and reduce waste.

## 5. Conclusions

PLHIV have a higher risk of mortality and ICU admission from COVID-19 compared to individuals without HIV. The higher mortality risk is more pronounced in individuals over 60 years of age. This conclusive evidence underscores the need for preventative measures and enhanced medical care for PLHIV co-infected with COVID-19, especially during outbreaks. The analysis indicates that effect sizes have stabilized, providing conclusive evidence of HIV’s impact on COVID-19-related mortality. Although further large meta-analyses may not significantly change this conclusion, future research remains valuable to assess evolving factors such as emerging SARS-CoV-2 variants, differential vaccine responses, and antiretroviral therapy-related influences. These findings also have important public health implications, highlighting the need for continued vaccine prioritization, enhanced monitoring, and tailored clinical management strategies for PLHIV during ongoing and future outbreaks.

## Figures and Tables

**Table 1:** Subgroup analyses by age group for COVID-19 mortality.

Outcome	Mortality	
Age Groups	Effect Size (95%CI), I^2^	Number of Studies
≤60	1.15 (0.13–2.17), 54%	4
>60	1.37 (0.08–1.93), 36%	3

**Table 2:** Meta-analysis by region for COVID-19 outcomes.

Outcome	Mortality		Hospitalization		ICU	
Region	Effect Size (95%CI), I^2^	Number of Studies	Effect Size (95%CI), I^2^	Number of Studies	Effect Size (95%CI), I^2^	Number of Studies
sub-Saharan Africa (aHR)	1.35 (0.65–2.06), 78.8%	8	NR	NR	1.59 (1.10–2.31), 0%	1
sub-Saharan Africa (aOR)	1.40 (0.81, 1.99), 54.6%	4	1.00 (0.63–1.57)	1	2.90 (1.20–6.90)	1
Europe (aHR)	1.75 (1.32–2.18), 31.3%	5	1.81 (0.90–2.73), 89.4%	2	1.29 (0.79–1.78), 0%	2
Europe (aOR)	1.13 (0.81–1.44), 0%	6	1.31 (0.21–2.42), 91.7%	2	1.38 (1.07–1.85)	3
Asia (aHR)	1.49 (0.99–2.24)	1	NR	NR	NR	NR
Asia (aOR)	1.77 (1.05–2.97)	1	NR	NR	NR	NR
North America (aHR)	1.04 (0.68–1.40), 99%	4	4.10 (1.40–12.40)	1	1.30 (1.80–4.41), 94.0%	2
North America (aOR)	1.39 (0.91–1.86), 95%	14	1.09 (0.75–1.43), 98%	8	1.46 (0.47, 2.45)	5
South America (aHR)	1.36 (1.13–1.63)	1	NR	NR	NR	NR
South America (aOR)	6.97 (1.92–25.28)	1	NR	NR	NR	NR

Confidence interval: CI; Intensive care unit: ICU; Not reported: NR.

## Data Availability

No new data were created or analyzed in this study.

## Supplementary Materials

The following supporting information can be downloaded at: https://www.mdpi.com/article/10.3390/medsci13040261/s1, Figure S1. Quality assessment of included primary studies. Figure S2. Leave-one-out analysis for COVID-19 Mortality. Figure S3. Publication bias assessment for the outcome of mortality. Figure S4. Publication bias assessment for the outcome of ICU. Figure S5. Leave-one-out analysis for COVID-19 ICU admission. Figure S6. Publication bias assessment for COVID-19 hospitalization. Figure S7. Leave-one-out sensitivity analysis for COVID-19 hospitalization. Table S1 Characteristics of included meta-analyses. Table S2 Characteristics of included primary studies.
