# Supplementary material for: Exit Meta-Analysis on the Effect of HIV on COVID-19 Mortality, Hospitalization, and ICU Admission
Source: Med Sci (Basel). 2025 Nov 7;13(4):261. doi: 10.3390/medsci13040261 (PMC12641877; doi:10.3390/medsci13040261)
Supplement: Supplementary file 1 [file medsci-13-00261-s001.zip › medsci-3924301-supplementary.pdf]

Systematic Review

Exit Meta-Analysis on the Effect of HIV on COVID-19 Mortality, Hospitalization, and ICU Admission

PubMed search

Search #1 Search terms for Covid-19 (n=1 009 661)

"covid 19" OR "covid19" OR coronavirus OR "coronaviruses" OR "novel" OR "novel s" OR "novels" OR "severe acute respiratory syndrome coronavirus 2" OR "severe acute respiratory syndrome coronavirus 2" OR "sars cov 2" OR "COVID" OR ("coronaviurs" AND "disease") OR ("2019-nCoV" AND "acute" AND "respiratory" AND "disease") OR ("Novel" AND "coronavirus" AND "pneumonia") OR ("Wuhan" AND "pneumonia") OR ("SARS-Cov-2")

Filters from 2019-2024

Search #2 Search terms for HIV (n=103 465)

"HIV" OR "Hiv" OR "hiv" OR "Aids" OR "human immunodeficiency virus " OR "human immunodeficiency virus " OR "acquired immunodeficiency syndrome" OR "acquired immunodeficiency syndrome" OR "ARV" OR "arv" OR "antiretroviral" OR "controlled HIV" OR "uncontrolled HIV"

Filters from 2019-2024

Search #3 - #1 AND #2 (n= 15 074)

Search #4- Search #3 restricted by meta-analysis article type (n = 109)

Cochrane reviews

Search #1 Search terms for Covid-19 (n=1 581)

"covid 19" OR "covid19" OR coronavirus OR "coronaviruses" OR "novel" OR "novel s" OR "novels" OR "severe acute respiratory syndrome coronavirus 2" OR "severe acute respiratory syndrome coronavirus 2" OR "sars cov 2" OR "COVID" OR ("coronaviurs" AND "disease") OR ("2019-nCoV" AND "acute" AND "respiratory" AND "disease") OR ("Novel" AND "coronavirus" AND "pneumonia") OR ("Wuhan" AND "pneumonia") OR ("SARS-Cov-2")

Search #2 Search terms for HIV (n=2 989)

"HIV" OR "Hiv" OR "hiv" OR "Aids" OR "human immunodeficiency virus " OR "human immunodeficiency virus " OR "acquired immunodeficiency syndrome" OR "acquired immunodeficiency syndrome" OR "ARV" OR "arv" OR "antiretroviral" OR "controlled HIV" OR "uncontrolled HIV"

Search #3 - #1 AND #2 (n= 723)

Search #4- Search #3 restricted by timeframe of 2019-2024 (n = 497)

EMBASE

Search #1 Search terms for Covid-19 (n= 2 681 448)

"covid 19" OR "covid19" OR coronavirus OR "coronaviruses" OR "novel" OR "novel s" OR "novels" OR "severe acute respiratory syndrome coronavirus 2" OR "severe acute

respiratory syndrome coronavirus 2" OR "sars cov 2" OR "COVID" OR ("coronaviurs" AND "disease") OR ("2019-nCoV" AND "acute" AND "respiratory" AND "disease") OR ("Novel" AND "coronavirus" AND "pneumonia") OR ("Wuhan" AND "pneumonia") OR ("SARS-Cov-2")

Search #2 Search terms for HIV (n=729 232)

"HIV" OR "Hiv" OR "hiv" OR "Aids" OR "human immunodeficiency virus " OR "human immunodeficiency virus " OR "acquired immunodeficiency syndrome" OR "acquired immunodeficiency syndrome" OR "ARV" OR "arv" OR "antiretroviral" OR "controlled HIV" OR "uncontrolled HIV"

Search #3 - #1 AND #2 (n= 50 296)

Search #4- Search #3 restricted by timeframe of 2019-2024 (n = 24 060)

Search #5- Search #4 limited by type of EBM as [cochrane review] OR [systematic review] OR [meta analysis] (n=869)

Table S1. – Characteristics of included meta-analyses.

Study	Number of studies	Adjusted or Unadjusted	Effect Size hospitalized	Effect Size Death	Effect Size ICU	Limitations
Dzinamarira, 2022	16 studies	unadjusted	Not reported	RR: 1.07 [0.86-1.32]	Not reported	-No bias adjustment in the model.<br>-No hospitalization and ICU assessed<br>- Unadjusted effect size (confounding)
Mellor, 2021	5 studies	adjusted	Not reported	HR: 1.95 [95% CI 1.62-2.34]	Not reported	-Small sample size of observational studies<br>-use of unadjusted effect sizes
Dong, 2021	10 studies	unadjusted	Not reported	OR: 1.25 (95% CI 1.027–1.524)	Not reported	-No bias adjustment in the model.<br>-No hospitalization and ICU assessed<br>- Unadjusted effect size (confounding)
Moradi, 2022	12 studies (11 reported mortality, 4 ICU)	unadjusted	Not reported	RR: 1.37 (95% CI 1.30-1.45)	RR: 1.24 (95% CI 1.08-1.41)	-No bias adjustment in the model<br>- Unadjusted effect size (confounding)<br>- Only 4 studies reported data for ICU
Danwang, 2022	23 studies included in mortality analysis.<br>6 included in hospitalization analysis	unadjusted	OR: 1.49; (95% CI 1.01–2.21)	OR: 0.81 (95% CI 0.47-1.41)	Not reported	-No bias adjustment in the model<br>- Unadjusted effect size (confounding)
	2 included in adjusted meta-analysis	Adjusted	Not reported	HR: 1.76 (95% CI 1.31–2.35)	Not reported	-No bias adjustment in the model<br>-Small sample size

Sarkar, 2021	7 studies	Unadjusted	Not reported	RR: 0.99 (95% CI 0.82-1.19	Not reported	-No bias adjustment in the model<br>-No hospitalization and ICU assessed<br>8- Unadjusted effect size (confounding)
Mousavi & Moosazadeh, 2023	4 studies included in mortality analysis.<br>3 studies included in hospitalization analysis	Unadjusted	OR: 1.67 (95% CI 0.76, 3.71)	OR: 0.80 (95% CI 0.57, 1.11)	Not reported	-Use of random effect model<br><br>-Did not report covariate adjusted effect measures
Favara, 2022	28 studies	Unadjusted	Not reported	OR: 1.09 (0.93-1.26)	Not reported	-No bias adjustment in the model.<br>-No hospitalization and ICU assessed<br>Unadjusted effect size (confounding)
Ssentongo, 2021	4 studies (2 for ICU analysis)	Unadjusted	Not reported	RR: 1.78 (1.21-2.60)	RR: 1.50 (0.84 - 2.67)	-No bias adjustment in the model.<br>-No hospitalization and ICU assessed<br>- Unadjusted effect size (confounding)<br>-Small sample size
Liang, 2021	14 studies<br><br>6 in meta-analysis	Unadjusted	Not reported	RR: 0.96 (0.88-1.06)	Not reported	-No bias adjustment (confounding)<br>-use of risk ratios not appropriate for meta-analysis<br>12
Kouhpayeh, 2021	11 studies	Unadjusted	Not reported	RR: 1.21 (1.02-1.43)	Not reported	-use of risk ratios not appropriate for meta-analysis
Wang, 2020	Overall, 7 studies in unadjusted analysis<br><br>3 studies included in adjusted analysis	Adjusted	Not reported	OR: 1.02 (95% CI 0.90-1.16)<br>aOR: 2.07 (1.73-2.47)	Not reported	-No hospitalization and ICU assessed<br>- use of risk ratio for meta-analysis is not recommended
Wang, 2021	84 studies	Unadjusted	Not reported	OR: 1.23 (1.02-1.48)	Not reported	-Included case series , and cross-sectional design
Wang, 2022	12 studies in unadjusted meta-analysis	Unadjusted	Not reported	Crude RR: 1.28 (0.98-1.69)	Not reported	-use of risk ratios not appropriate for meta-analysis
	16 studies in adjusted meta-analysis	Adjusted	Not reported	ARR: 1.30 (1.09-1.56)	Not reported	-use of risk ratios not appropriate for meta-analysis
Oyelade, 2022	43 studies (31 in meta-analysis)	Unadjusted	Not reported	RR: 1.5 (1.45 – 2.03)	Not reported	-Use of unadjusted effect measures<br>No assessment of hospitalization or ICU admission.

Han, 2023	40 studies	Adjusted	Not reported	Adjusted Pooled effect (aOR,aRR ,aHR) : 1.43 (1.25 – 1.63)	Not reported	-Combined Effect sizes reported with no stratification by type of effect sizes (aOR, aRR, aHR) -No use of quality assessment in meta-analysis
Hariyanto, 2021	28 studies	Unadjusted	Not reported	OR: 1.19 (1.01 – 1.39)	Not reported	No adjustment for confounders
Baluku, 2020	6 studies	Unadjusted	Not reported	Mortality rate among PLHIV: 0.09 (0.03-0.15)	Not reported	-Small number of primary studies -included surveys, case-series, case-reports and commentaries -No use of quality assessment within meta-analysis -Reported Pooled mortality rate among PLHIV with COVID-19, without comparison group.
Lee, 2020	7 studies	Unadjusted	Not reported	Mortality rate among PLHIV with COVID-19 coinfection : 5.3%	Not reported	-Reported Pooled mortality rate among PLHIV with COVID-19, without comparison group.
Treskova-Schwarzbach,2021	9 studies (1 study for ICU)	Unadjusted	Not reported	From OR 1.7 (1.32-2.18) in Africa to OR 0.07 (0.03-0.52) in North America	OR 1.22 (0.8-1.87)	-Very few studies for each outcome -Use of unadjusted effects sizes

Meta-analyses were defined as adjusted if they used adjusted effect size in their synthesis, and unadjusted if they extracted raw numbers of participants with and without a particular outcome and synthesized them in meta-analysis.

Table S2. – Characteristics of included primary studies.

Study ID	Study design	Country	Period data collection	Mean age ± SD or Median (IQR)	% Female	Outcomes reported	Model used for adjustment
Adrish, 2020	Retrospective cohort	USA	Mar-20	64.6 ± 12.13	-	ICU	Age and comorbidities
Ahmed,2022	Cohort	Ethiopia	Mar - Oct 2021	49.5(40-59) HIV, 48(32-65) no HIV	39%	Mortality	Model 2 + symptoms

Bennett, 2021	Retrospective cohort	Ireland	Mar- Jul 2020	Not reported	.	Mortality, hospitalization and ICU	Age
Bergman, 2021	Retrospective cohort	Sweden	Jan - Sep 2020	46 ± 21	60.9%	Mortality, hospitalization and ICU	.
Bertagnolio,2022	Case-control	Multiple countries	Not reported	45.5 ± 13.7	53%	Mortality	Model 2+ disease severity at admission
Bhaskaran, 2021	Retrospective cohort	UK	Feb - Jun 2020	Not reported	35.3%	Mortality	Model 1
Boswell,2023	Cohort	South Africa	Apr - Nov 2020	54 ± 16	50%	In-hospital mortality	Age
Brown, 2022	Retrospective cohort	England	Mar - Jun 2020	.	31%	Mortality	Model 4
Bushman, 2021	Case-control	USA	Mar - Apr 2020	56 (23-64)	34.5%	Mortality	.
Cai, 2021	Cohort	USA	Mar - Sep 2020	63.3 (49.8–73.1)	11.5%	30 days mortality	Model 5 + contextual factors, and COVID-19 testing capacity and hospital occupancy.
Chanda, 2021	Case-control	Zambia	Mar - Dec 2020	46.4 (12.9)	48%	Mortality and hospitalization	Model 2+ treatment center
Deiana, 2020	Case-control	Italy	Until April 2020	50−59	59.2%	Mortality	Model 1
Durstenfeld, 2022	Retrospective cohort	USA	Apr - Dec 2020	56.0 ± 13.0	27.7%	Mortality	Model 5 + insurance
Emami, 2020	Retrospective cohort	Iran	Feb - Mar 2020	.	.	Mortality	Age and comorbidities
Filardo, 2020	Retrospective cohort	USA	Mar - Apr 2020	.	32.6%	Mortality	Model 5
Fleischer, 2022	Retrospective cohort	Kampala, Uganda	Apr - Jul 2021	54(16)	51%	In-hospital mortality and ICU	Model 1
Gagliardini, 2021	Retrospective cohort	Italy	Jan 2020 - Jun 2021	61(50-71)	33.4%	Mortality and ICU	Model 2 + ratio of arterial oxygen partial pressure to fractional inspired oxygen (PaO2/FiO2) and pneumonia at admission to the hospital
Ge, 2021	Retrospective cohort	Canada	Jan - Dec 2020	42.7	52%	30 day all-cause mortality	Model 5 + socioeconomic variables

Geretti, 2020	Cohort	England, Scotland, and Wales	Jan - Jun 2020	56 (49 - 62) in PLWH	33.9%	Mortality and ICU	Model 5 + indeterminate/probable hospital acquisition of COVID-19
Guo, 2023	Retrospective cohort	USA	Jan 2020 - Oct 2021	NM	54.9%	Mortality and hospitalization	Model 5
Hadi, 2020	Retrospective cohort	USA	NM	48.2 years (SD 14.2) in PLWH	29% in PLWH	Mortality and hospitalization	Model 3 without age + history of nicotine dependence
Hedberg, 2022	Cohort	Sweden	Mar 2020 - Aug 2021	44	52%	All cause-mortality and hospitalization	Model 2 + the month of positive SARS-CoV-2 test
Jassat, 2021	Cohort	South Africa	Mar 2020 - Mar 2021	54 (40–66)	21.7%	Mortality	Model 5 + health sector, province, month of admission, and past or current Tb
Kabarrti, 2020	Retrospective cohort	USA	Mar - Apr 2020	.	53%	Mortality	Model 2 + socioeconomic status
Kaplan-Lewis, 2021	Retrospective cohort	USA	Mar - Apr 2020	55.2 ± 13.7	30%	Mortality and ICU	Model 5
Kelly, 2021	Cohort	USA	Mar - Sep 2020	.	.	Mortality	Model 4 + race, marital status, clinical factors, and month of COVID-19 diagnosis.
Laracy, 2021	Retrospective cohort	USA	Mar - Jun 2020	58.4 ± 12.7	29%	Mortality, hospitalization and ICU	.
Lea,2023	Cohort	USA	Mar - Nov 2020	50-59	54%	Mortality and hospitalization	Model 5 + BMI, smoking status, calendar period, and CD4 count and HIV viral load
Lee, 2021	Retrospective cohort	UK	Feb - May 2020	57 (50–63)	38.2%	Mortality	.
Li, 2021	Cohort	USA	Feb - Mar 2020	66 (58, 75)	47.8%	Mortality	Model 3 + hospital stay duration, smoking status, vital signs, ICU stay, WBC, creatinine, anion gap,

							potassium, and ALT.
Looha, 2022	Retro-spective cohort	Iran	Mar 2020 - Jan 2021	58	52.8%	Mortality and ICU	Model 2 + partial pres-sure of oxy-gen (PO2) < 93,
Lundon, 2020	Cross-sectional	USA	Mar - Apr 2020	58.0 ± 18.8	39%	Mortality and hospi-talization	Model 5 + smoking sta-tus
Makker, 2021	Retro-spective Cohort	USA	Feb - Apr 2020	61 ± 14	.	Mortality	Co-morbidi-ties, GI bleeding, steroids use, mechanical ventila-tion,LDF, D-Dimer, CRP levels
Mascarello, 2021	Cross-sectional	Brazil	Feb - Sep 2020	.	53%	Mortality, hospitali-zation, and ICU	Model 1 + race and schooling
Minchella, 2022	Cohort	Zambia	Mar 2020 - Feb 2022.	50.6 [19.5]	45.7%	Mortality	Model 2 + wave (1-4), disease sta-tus at admis-sion, COVID-19 vaccina-tion status, and COVID-19 treatment center
Mphekgwana, 2023	Retro-spective cohort	South Africa	Mar 2020 - Jun 2021	.	.	COVID-19 mortality within 24 hours of hospital admission	Sex
Nyasulu, 2022	Prospec-tive co-hort	South Africa	Mar 2020 - Nov 2020	54.1 (46.0–61.6)	45%	Mortality and ICU	Model 2 + smoking sta-tus, symp-toms of initial presentation, arterial blood gas,
Orlando, 2021	Retro-spective cohort	Italy	Covid-19 registry un-til 10 Jun 2020	.	44.4%	Mortality	NM
Osibogun, 2021	Retro-spective cohort	Nigeria	Feb 2020 - Jul 2020	43 years (IQR: 33–55)	34.2%	Mortality	Model 1
Parker, 2022	Retro-spective cohort	South Africa	Mar 2020 - Jul 2020	HIV-unin-fected:54 (43–65) PWH:46 (39–52)	57%	Mortality	Model 3
Paul,2021 (rural)	Cohort	USA	as of Octo-ber 23, 2020	.	50%	Mortality	.

Paul, 2021 (urban)	Cohort	USA	as of October 23, 2020	.	50%	Mortality	.
Perez-guzman, 2021	Cohort	England	Feb - May 2020	69 (IQR = 25)	38%	In hospital Mortality	Age
Robles-Perez, 2021	Cohort	Mexico	Mar - Dec 2020	37.07 years	56.7%	Mortality	Model 2
Rosenthal, 2022	Retrospective cohort	USA	Mar - Jun 2020	.	29%	In hospital-mortality and ICU	Model 2 + admitting facility, and admission date
Semenzato, 2021	Retrospective cohort	France	Feb - Jul 2020	67 ± 19	46.7%	Mortality	Model 1
Sigel, 2020	Retrospective cohort	USA	Mar - Apr 2020	61 (54–67) IN PLWH	25% in PLWH	Mortality	Model 4 + COPD, smoking, baseline ferritin level, and baseline WBC
Sohrabi, 2021	Retrospective cohort	Iran	Mar - Dec 2020	52.8	47.4%	Mortality	NM
Spence,2022	Retrospective cohort	USA	Jan - Nov 2020	51.2	37.0%	Mortality and mechanical ventilation	Model 2 + insurance
Sun, 2021	Cohort	USA	Jan 2020 - May 2021	47(32-61)	55%	Mortality, hospitalization, and ICU	Model 4 + smoking status, and study site.
Tang, 2022	Retrospective cohort	USA	Mar- Nov 2020	NM	.	In hospital mortality, hospitalization, and ICU	Model 3 + race
Tesoriero, 2021	Retrospective cohort	USA	Mar - Jun 2020	54.0 ± 13.0	29.4%	Mortality and hospitalization	Model 1 + region
Turtle, 2023	Prospective cohort study	UK	Jan 2020 - Feb 2022	Immunocompetent: 69.5 (53.4 to 82.0) HIV+: 57.8 (49.8 to 70.5)	44.6%	Mortality, ICU, mechanical ventilator	Model 4 + deprivation index, vaccination, and comorbidities
Venturas, 2021	Cohort	South Africa	Mar - Sep 2020	50 (39–60)	47%	Mortality and hospitalization	.
WCD-NIH, 2022	Cohort	South Africa	Mar to Jun 2020	63	58%	Mortality	Model 2
Yang, 2021	Cohort	USA	Jan 2020 - May 2021	47 (32-60)	54.9%	Mortality and hospitalization	Model 4 + race
Yendewa, 2021	Retrospective cohort	USA	Jan - Dec 2020	48.34 ± 13.59	30.6% in PLWH	30-days mortality, hospitalization and ICU	Model 5

Zimmer-mann, 2021	Retro-spective cohort	Brazil	Mar - Oct 2020	44.52%	NM	Mortality	NM

Model 1: age and sex; Model 2: age, sex, and comorbidities; Model 3: age, sex, obesity or BMI, and comorbidities; Model 4: age, sex, and ethnicity; Model 5: age, sex, race and comorbidities; ICU: intensive care unit; NM: not mentioned; NYC: New York City; PLWH: people living with HIV, SD: standard deviation UK: United Kingdom; USA; United States of America.

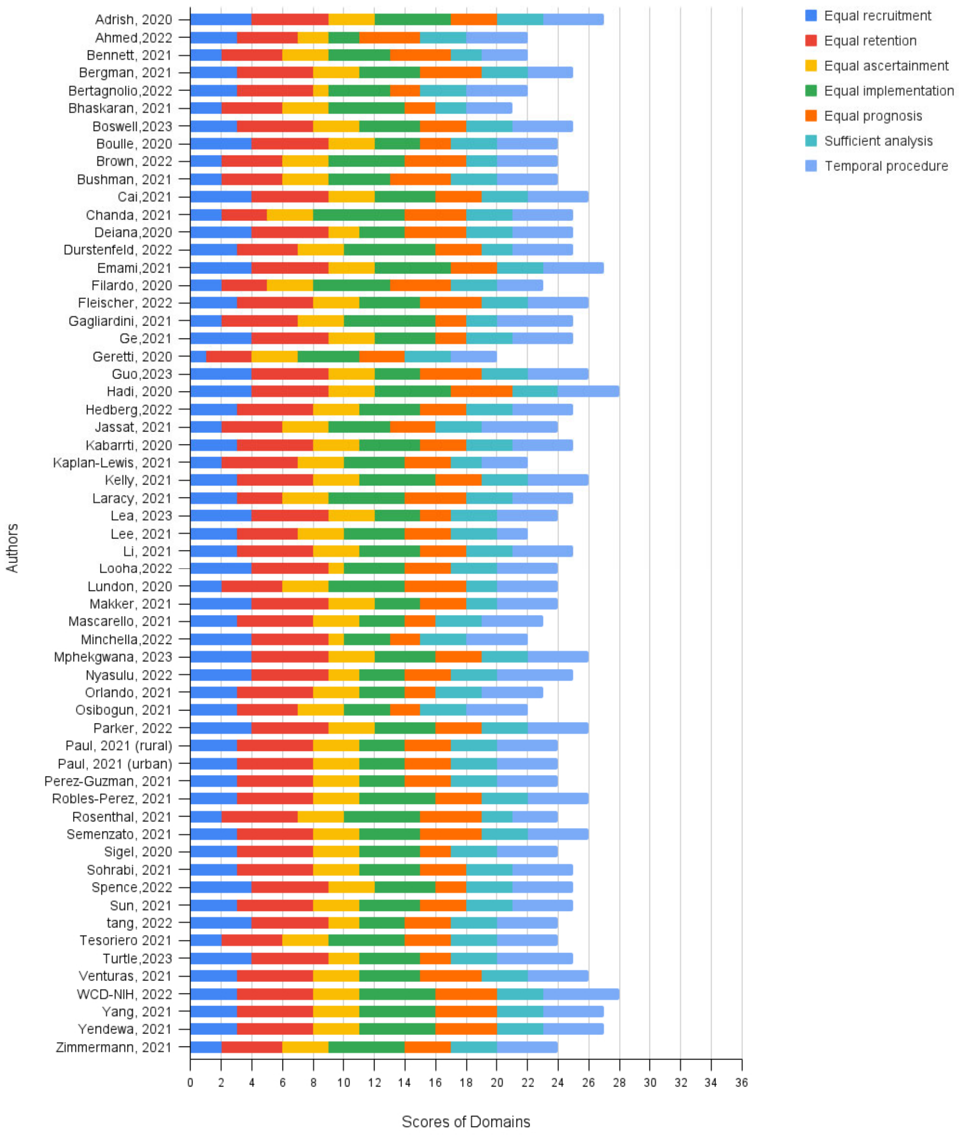

Supplementary Figure S1

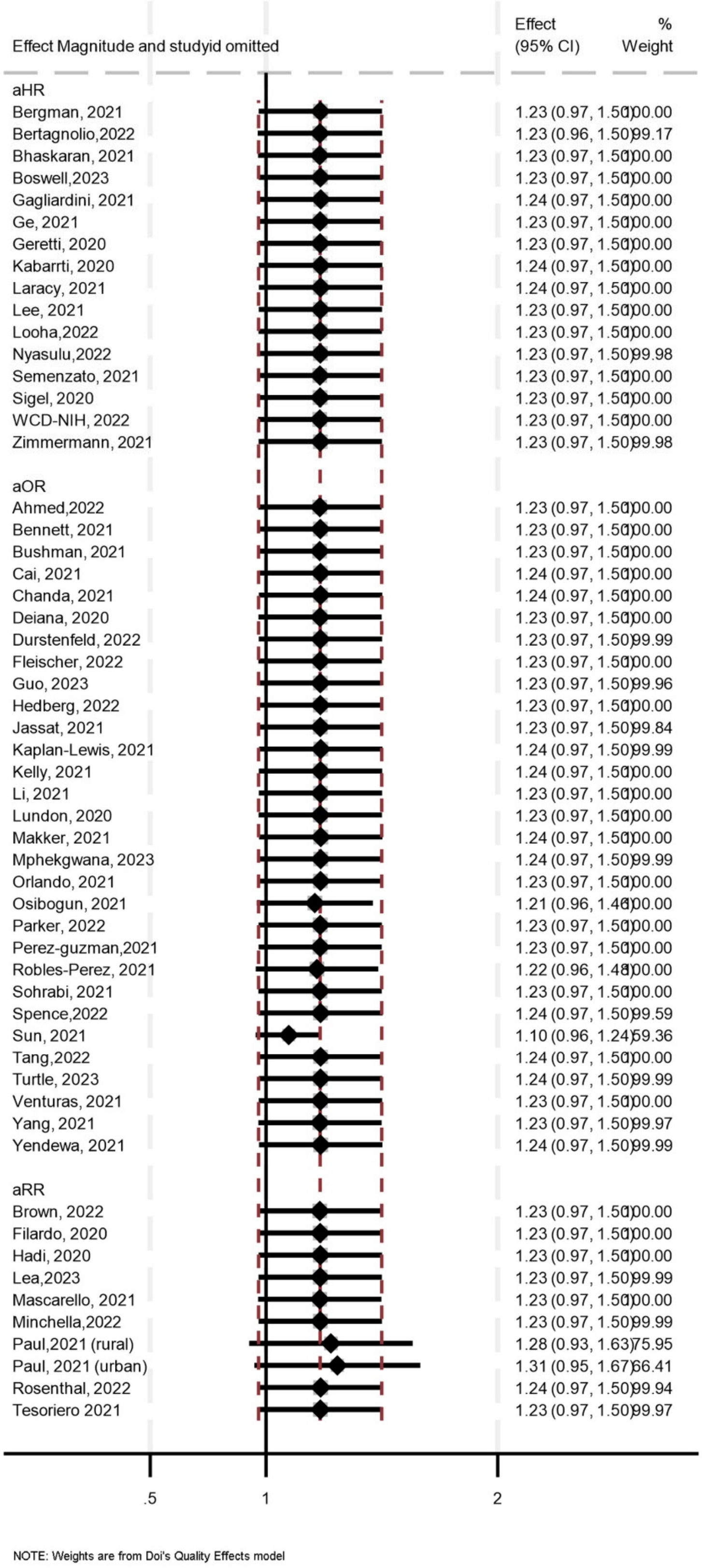

Supplementary Figure S2.

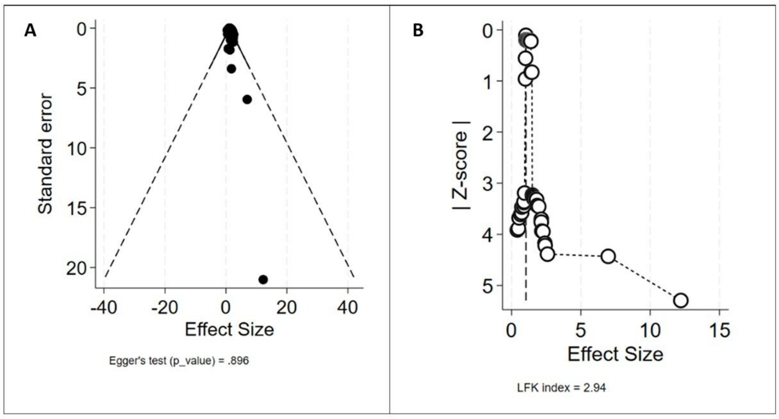

Supplementary Figure S3

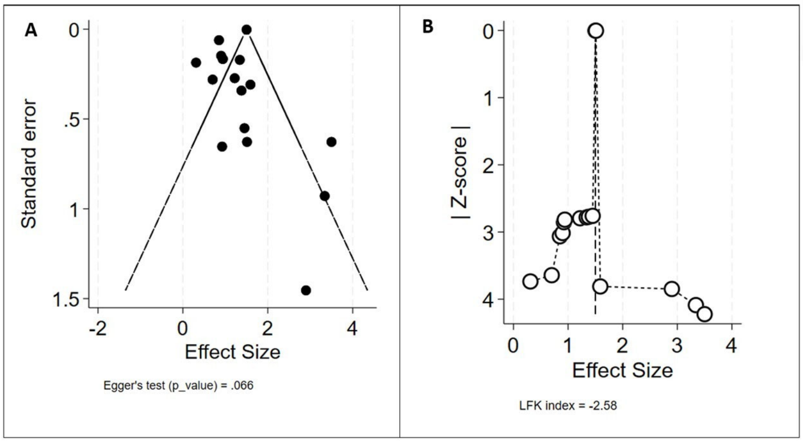

Supplementary Figure S4

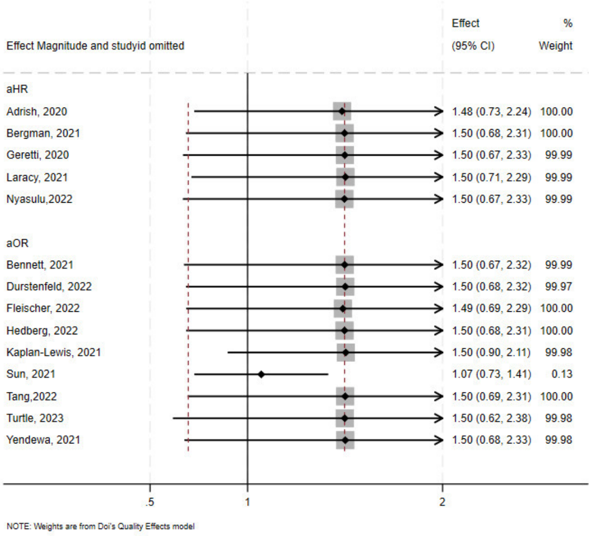

Supplementary Figure S5

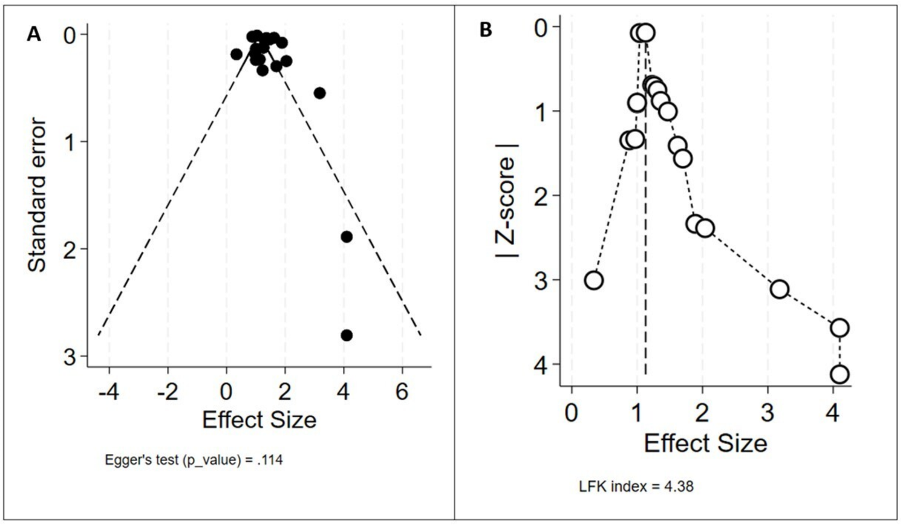

Supplementary Figure S6

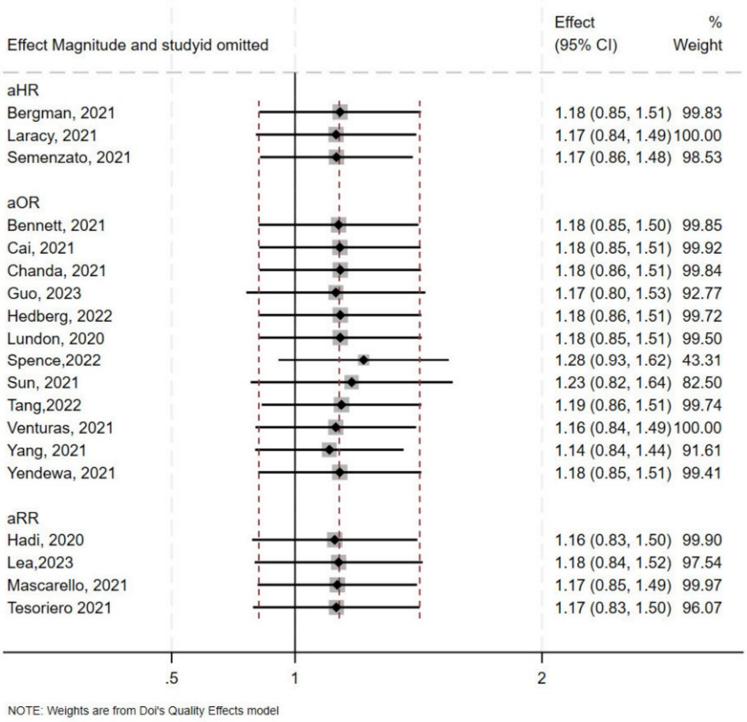

Supplementary Figure S7.
